# Baicalin inhibits TLR7/MYD88 signaling pathway activation to suppress lung inflammation in mice infected with influenza A virus

**DOI:** 10.3892/br.2014.253

**Published:** 2014-03-14

**Authors:** QIAOFENG WAN, HAO WANG, XUEBO HAN, YUAN LIN, YANHUI YANG, LIGANG GU, JIAQING ZHAO, LI WANG, LING HUANG, YANBIN LI, YURONG YANG

**Affiliations:** 1 Department of Pathogenic Biology and Immunology, Basic Medical Science College, Ningxia Medical University, Yinchuan, Ningxia 750004, P.R. China; 2 Key Laboratory of Antivirus of the Ministry of Education, Beijing University of Chinese Medicine, Beijing 100029, P.R. China

**Keywords:** baicalin, influenza A virus, lung, TLR7/MYD88, c-jun/activator protein 1, nuclear factor κB

## Abstract

The present study aimed to investigate the protective effects and underlying mechanisms of baicalin on imprinting control region mice infected with influenza A/FM/1/47 (H1N1) virus. Oral administration of baicalin into mice infected with H1N1 prevented death, increased the mean time to death and inhibited lung index and lung consolidation. Subsequently, fluorescence quantitative polymerase chain reaction was used to assess the mRNA expression of toll-like receptor 7 (TLR7) and myeloid differentiation primary response gene 88 (MYD88), and western blot analysis was used to determine the expression of phosphorylated nuclear factor κB (NF-κB)-P65 and c-jun/activator protein 1 (AP-1). An enzyme-linked immunosorbent assay was applied to test for the inflammatory cytokines, tumor necrosis factor (TNF)-α and interleukin (IL)-1β and IL-6, in the lung tissue. The findings indicated that baicalin downregulated the mRNA expression of TLR7 and MYD88, significantly downregulated the protein expression of NF-κB-P65 and AP-1 and also inhibited the secretion of TNF-α, IL-1β and IL-6. In conclusion, baicalin effectively reduced the pathological damage and inflammation of the lungs by downregulating the TLR7/MYD88-mediated signaling pathway.

## Introduction

Influenza is an acute respiratory disease, with high morbidity and mortality rates in humans and animals (1). The influenza A virus is prone to antigenic drifting or antigenic conversion and reorganization of the genome, which results in the emergence of novel subtypes and a lack of immunity in the majority of the population (2). The Western medicine currently available for the prevention and treatment of influenza A virus infection causes severe side-effects and drug resistance due to the wide range of applications (3,4). To date, the vaccines against the latest influenza A virus have been ineffective. Therefore, traditional Chinese medicine is beginning to play an important role against influenza A virus infection, which could be mainly regulated by the immune system (5).

Baicalin is a flavonoid extracted from the root of *Scutellaria baicalensis* that demonstrates a variety of biological activities (6–8). Baicalin has been found to be an inhibitor of the reverse transcriptase of the human immunodeficiency virus *in vitro* (9). Baicalin also acts as a potent inhibitor of the hepatitis B virus by reducing DNA synthesis (10). In addition, baicalin has been reported to have protective effects and inhibit death in mice infected with influenza A virus (11). Currently, there are no available studies on the antiviral mechanisms of baicalin against the influenza A virus.

The aim of the present study was to investigate the effects of baicalin on the mRNA expression of toll-like receptor 7 (TLR7) and myeloid differentiation primary response gene 88 (MYD88), and the effects on the protein expression of phosphorylated nuclear factor κB (NF-κB)-P65 and c-jun/activator protein 1 (AP-1), together with the expression of interleukin (IL)-1β, tumor necrosis factor (TNF)-α and IL-6, in the lungs of mice infected with influenza A/FM1/1/47 (H1N1), in order to determine the mechanisms underlying the antiviral effects of baicalin and to provide evidence *in vitro* that may result in the development of novel anti-influenza drugs.

## Materials and methods

### Mice and H1N1

Two batches of imprinting control region (ICR) mice (n=216), weighing 13–15 g, were treated according to the ‘Guide for the Care and Use of Laboratory Animals’ prepared by the National Institutes of Health. The first and second batches consisted of 120 (qualification no. 0213824) and 96 mice (qualification no. 0218801), respectively, and were purchased from the Vital River Experimental Center (Beijing, China). H1N1 was provided by the China Academy of Traditional Chinese Medicine (Beijing, China) and was transplanted into the allantoic cavity of 9-day-old embryonated hen eggs 3 times in succession. This study was approved by the Ethics Committee of the Beijing University of Chinese Medicine (Beijing, China).

### Drugs

Baicalin was provided by Professor Zhengyun Chu from the Liaoning Traditional Chinese Medicine University (Shenyang, China). Ribavirin particles were purchased from Sichuan Bali Pharmaceutical Co., Ltd. (Chengdu, China).

### Reagents

An M-MLV reverse transcription kit (Takara Bio, Inc., Shiga, Japan), TaqE (Takara), dNTP (Takara), DNA marker (Takara), SYBR-Green mix (Bio-Rad, Hercules, CA, USA), agarose (Promega Corporation, Madison, WI, USA), diethylpyrocarbonate (Sigma, St. Louis, MO, USA) and TRIzol (Invitrogen Life Technologies, Carlsbad, CA, USA) were used in this study. Primers were synthesized by Sangon Co., Ltd. (Shanghai, China) as follows: GAPDH (201 bp) forward, 5′-CTCATGACCACAGTCCATGC-3′ and reverse, 5′-CACATTGGGGGTAGGAACAC-3′; TLR7 (117 bp) forward, 5′-ACGCTTTCTTTGCAACTGTG-3′ and reverse, 5′-TTTGTGTGCTCCTGGACCTA-3′; and MYD88 (136 bp) forward, 5′-TGGTGGTTGTTTCTGACGAT-3′ and reverse, 5′-GGAAAGTCCTTCTTCATCGC-3′. NF-κB-P65 and p-c-jun rabbit monoclonal primary antibodies were purchased from Bioworld Technology Co., Ltd (Nanjing, China), while IgG goat polyclonal secondary antibody was purchased from the Beijing Zhongshan Golden Bridge Biotechnology Co., Ltd. (Beijing, China) and cell lysis buffer was bought from Beyotime (Nanjing, China). The western blot analysis was conducted at the Key Laboratory of Antiviru of the Ministry of Education. Enzyme-linked immunosorbent assay (ELISA) kits, IL-1β, TNF-α and IL-6 were obtained from Bender MedSystems (Vienna, Austria).

### Effects of baicalin on mouse survival

The ICR mice (n=120) were randomly divided into 6 groups, as presented in Table I. The mice were lightly anesthetized by the inhalation of diethyl ether and intranasally infected with 10X LD50 of H1N1, except the normal group who received sodium chloride. The mice were treated with baicalin at various doses (93.75, 187.5 and 375 mg/kg/day); the ribavirin group was used as a positive control and received ribavirin (100 mg/kg/day), and the placebo and normal groups were treated with sodium chloride (200 μl). All the mice underwent oral gavage once daily for 7 days at the beginning of the study, 24 h post-virus inoculation. Each group was observed for 14 days and the number of deaths were recorded.

### Effects of baicalin on mouse lung parameter

The second batch of 96 ICR mice were randomly divided into 6 groups and treated as aforementioned. After 5 days of treatment, the mice were weighed and sacrificed by orbital blood, and the lungs were removed and weighed. The lung index and lung index inhibition were calculated as follows (12): Lung index = A / B × 100; and lung index inhibition = (C - D) / C × 100, where A is the lung weight, B is the body weight, C is the lung index of the placebo group and D is the lung index of the drug-treated group. Four lungs from each group were fixed in 10% formalin solution and then embedded in paraffin for histological examination. The remaining lungs were cut in half; one half was homogenized for ELISA and the other half was stored in liquid nitrogen for protein and total RNA extraction.

### mRNA assay of TLR7 and MYD88 by fluorescence quantitative polymerase chain reaction (qPCR)

Total RNA of the lung tissue was extracted by TRIzol reagent. The reverse transcription system (25 μl) was as follows: 3 μl RNA, 1 μl oligo(dT), 5 μl 5X buffer, 5 μl dNTP (10 mmol/l), 0.5 μl RNase inhibitor, 1 μl M-MLV and 9.5 μl ddH_2_O. The mixture was incubated at 42°C for 60 min and then at 70°C for 10 min. The fluorescence qPCR system (20 μl) was as follows: 1.5 μl cDNA, 0.5 μl primers F (10 pmol/μl), 0.5 μl primers R (10 pmol/μl), 10 μl SYBR-Green mix and 7.5 μl ddH_2_O. qPCR was performed as follows: Predenaturation at 94°C for 15 min, denaturation at 95°C for 15 sec, annealing at 62°C for 30 sec, extension at 72°C for 15 sec for a total of 40 cycles and then extension at 72°C for 10 min. The PCR products were assessed by electrophoresis in 1.2% agarose gel, and the integral optical density (IOD) value of GAPDH and the target bands were observed by the gel imaging analysis system (Beijing Binta Instrument Technology Co., Ltd, Beijing, China), with ethidium bromide staining. The ratio of the IOD value of the target bands to the IOD value of GAPDH was calculated as the relative expression of the target gene.

### Western blot analysis of NF-κB-P65 and c-jun/AP-1

Lung tissue was ground into a powder in liquid nitrogen, and protein was extracted using lysis buffer according to the manufacturer’s instructions. The protein was quantified by the Bradford protein assay. A total of 40 μg protein sample dissolved in 10 μl phosphate-buffered saline was added to an equal volume of 1X sample buffer. Subsequent to being boiled for 5 min, the proteins were separated by SDS-PAGE electrophoresis and transferred to polyvinylidene fluoride membranes using a semi-electric switch membrane machine at 30 mA for 90 min. The membranes were blocked by anti-NF-κB-P65 and anti-p-c-jun antibodies at 4°C overnight and then washed 3 times using Tris-buffered saline and Tween 20 buffer for 10 min each. Horseradish peroxidase-conjugated secondary antibodies were then added and oscillated at 37°C for 60 min. Electrochemiluminescence liquid was added and the samples were exposed to X-ray film after developing and fixing. The results were scanned for use after the observation.

### ELISA of TNF-α, IL-1β and IL-6

Lung tissue homogenates were prepared and ELISA was conducted according to the manufacturer’s instructions.

### Statistical analysis

The NF-κB-P65, c-jun/AP-1 and β-actin gray values were measured by Image-Pro Plus 6.0 software (Media Cybernetics, Inc., Rockville, MD, USA), and the ratio of the gray value was associated with the protein expression level. The assay data was analyzed by SPSS software, version 13.0 (SPSS Inc., Chicago, IL, USA). The group data are presented as the mean ± standard error of the mean. P≤0.05 was considered to indicate a statistically significant difference.

## Results

### Protective effects of baicalin on mice

**Figure 1:**
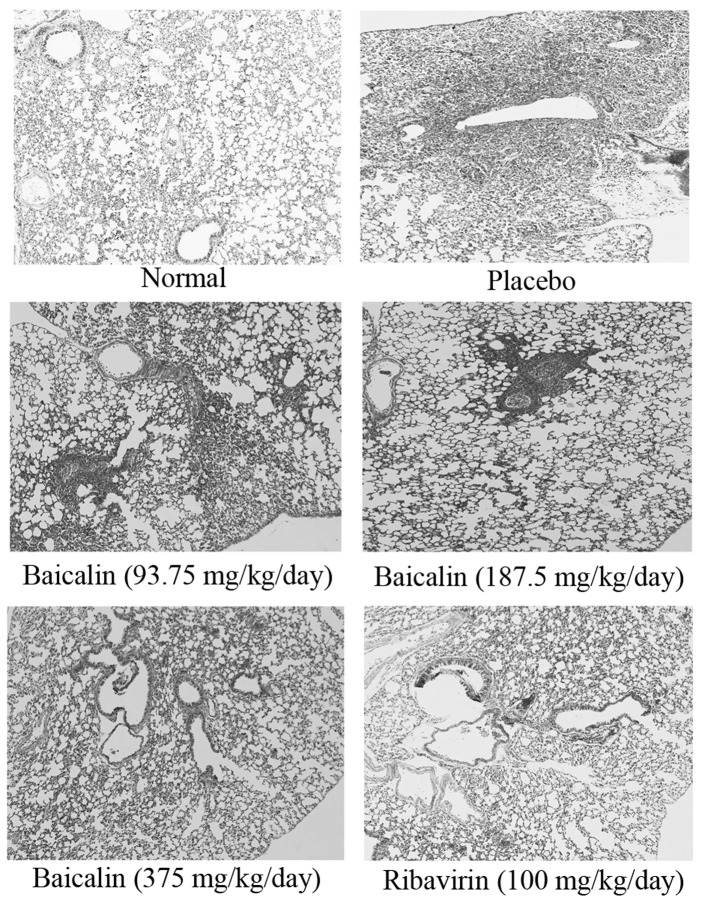
Effects of baicalin on the pathological characteristics of mice infected with influenza A/FM1/1/47 (hematoxylin and eosin stain; magnification, ×100). The mice were sacrificed 5 days after infection. The lungs were removed and washed with sterile phosphate-buffered saline. Subsequent to being fixed in 10% formalin and embedded in paraffin, the lungs were sectioned for histological examination. The pathological changes were evaluated based on hyperemia, bronchiole epithelium cell necrosis, lung exudates, alveolus interstitial pneumonia and lung abscesses.

Baicalin displayed a protective effect on mice with influenza A infection (Table I). All the doses of baicalin (93.75, 187.5 and 375 mg/kg/day) significantly prolonged the survival time of the mice. A survival rate of 55% was obtained at the lowest dose of 93.75 mg/kg/day. Compared with the other 2 doses, baicalin at a dose of 375 mg/kg/day exerted the best effects with a survival rate of 95% and a mean time to death (MDD) of 13.70±3.31 days. The lung parameter data showed that baicalin provided dose-dependent protective effects against viral pneumonia (Table I). Inhibition of the lung index was detected at 14.8, 32.4 and 34.5% at doses of 93.75, 187.5 and 375 mg/kg/day baicalin, respectively. The findings of the histological examination were consistent with the lung parameter data (Fig. 1). Protection from bronchitis and interstitial pneumonia was found in all the groups that were treated with baicalin, and the degrees of protection varied depending on the dose.

### Effects of baicalin on mRNA expression of TLR7 and MYD88

The method described by Livak and Schmittgen (13) was used to assess the effects of baicalin on the mRNA expression of TLR7 and MYD88 (Table II). Compared with the normal group, the mRNA expression levels of TLR7 and MYD88 were higher in the placebo group (P<0.01). The mRNA expression levels of TLR7 and MYD88 at doses of 187.5 and 375 mg/kg/day baicalin were significantly lower (P<0.01) compared with the placebo group.

### Effects of baicalin on the protein expression of NF-κB-P65 and c-jun/AP-1

**Figure 2:**
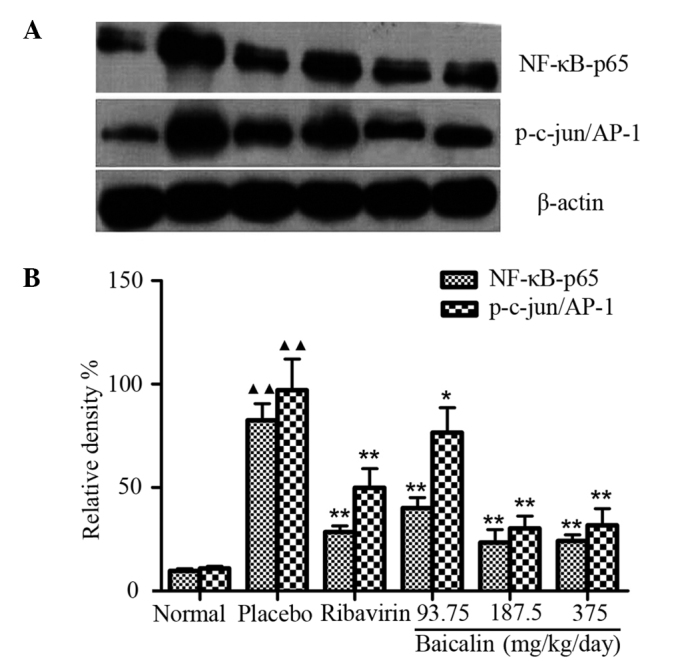
Effects of baicalin on the protein expression of NF-κB-P65 and c-jun/AP-1 by (A) western blot and (B) statistical data analyses. Western blot analysis was performed following the determination of the protein concentration by the Bradford protein assay and showed that compared with the normal group, the protein expression levels of NF-κB-P65 and c-jun/AP-1 were higher in the placebo group (P<0.01). The protein expression of NF-κB-P65 and c-jun/AP-1 at 187.5 and 375 mg/kg/day baicalin were lower (P<0.01) compared with the placebo group. ^▲▲^P<0.01 compared with the normal group; ^**^P<0.01 and ^*^P<0.05 compared with the placebo group. NF-κB-P65, nuclear factor κB-P65; AP-1, activator protein 1.

Compared with the normal group, the protein expression levels of NF-κB-P65 and c-jun/AP-1 were higher in the placebo group (P<0.01). The expression levels of NF-κB-P65 and c-jun/AP-1 at doses of 187.5 and 375 mg/kg/day baicalin were significantly lower (P<0.01) compared with the placebo group (Fig. 2).

### Effects of baicalin on the expression of TNF-α, IL-1β and IL-6

The expression levels of TNF-α, IL-1β and IL-6 were higher in the placebo group (P<0.01) compared with the normal group. The expression of TNF-α, IL-1β and IL-6 at doses of 187.5 and 375 mg/kg/day baicalin were significantly lower (P<0.01) compared with the placebo group (Table III).

## Discussion

In the present study, the inhibitory activity of baicalin against H1N1 in mice was examined and its mechanisms investigated. The oral administration of baicalin showed positive effects on the mice infected with H1N1, increasing the survival rate, prolonging the MDD and inhibiting lung index and lung consolidation.

The aim of the present study was to investigate the mechanisms underlying the antiviral activity of baicalin. It has been reported that the pathological injury caused by H1N1 infection is due to host inflammatory responses to the virus rather than the virus directly destroying respiratory epithelia (14). TLR, as a pattern recognition receptor, plays a role in recognizing the pathogen of influenza virus infection (15). TLR identifies extracellular and intracellular pathogen associations with the molecular pattern of the invading virus (16,17). TLR7 is activated once the single stranded RNA of influenza A is identified, and signals of the immune cells are transmitted through the MYD88 pathway. The NF-κB and AP-1 signaling pathways are then induced (18,19) resulting in the activation of cytokines, such as interferon-α/β, TNF-α, IL-1 and IL-6 production (20), which is a trigger of the subsequent antiviral acquired immunity reaction (21). When these pathways are out of control, a large number of pro-inflammatory mediators are produced causing inflammatory injury. Previous studies have shown that various levels of TNF-α, IL-1β and IL-6 are associated with the degree of pathological damage (22).

Morphology is closely associated with intracellular biochemical changes. In the present study, hematoxylin and eosin staining revealed severe pneumonia in the placebo group, including hyperemia, leukopedesis, bronchiole epithelium cell necrosis, lung exudates, alveolus interstitial pneumonia and lung abscess. Molecular biology showed that the mRNA expression of TLR7 and MYD88, the protein expression of NF-κB and AP-1 and the secretion of TNF-α, IL-1β and IL-6 were significantly increased in the placebo group. Following treatment with various doses of baicalin, the number of pulmonary lesions was reduced significantly, and the mRNA expression of TLR7 and MYD88, the protein expression of NF-κB and AP-1 and the secretion of TNF-α, IL-1β and IL-6 were significantly decreased, indicating that baicalin inhibits the activation of the TLR7/MYD88 signaling pathway to reduce the secretion of inflammatory cytokines, thereby reducing the inflammatory injury and restoring the stability and balance of immune function.

The signal transduction pathway induced by influenza virus infection is a complex network. Certain signal molecules play multiple roles following the infection of influenza virus, therefore it is difficult to determine a specific signal molecule to facilitate or inhibit the proliferation of the influenza virus. NF-κB is generally considered to be the key transcription factor that affects the secretion of type I interferon and other antiviral cytokines (23,24), but its moderate activation is a prerequisite for the infection of the influenza virus (25). It is known that the NF-κB pathway can regulate the synthesis of influenza virus RNA and that the knockout of P65 reduces RNA synthesis (26). In a sense, when studying NF-κB-P65 it is difficult to grasp the interactions between the influenza virus and its host as a whole. Therefore, the systematic methods based on the genome, the transcriptome, the proteome and bioinformatics may contribute to our understanding of the interpretation of influenza virus infection and antiviral drugs.

## Figures and Tables

**Table I:** Protective effects of baicalin on mice with influenza A infection (n=120).

				Lung parameters		
				
Group	Dose, mg/kg/day	Survival rate, %	MDD	Score	Lung index	Lung index inhibition, %
Normal	-	100.00	14.00±0.00	0.0±0.00	0.79±0.14	-
Placebo	-	5.00	7.25±2.82	3.2±0.71	1.42±0.31^a^	-
Ribavirin	100.00	100.00^b^	14.00±0.00^b^	1.2±0.53^c^	0.89±0.13^c^	37.3
Baicalin	93.75	55.00^b^	10.40±2.96^d^	2.5±0.65^d^	1.21±0.22^d^	14.8
	187.50	85.00^b^	13.05±3.08^c^	1.7±0.45^c^	0.96±0.21^c^	32.4
	375.00	95.00^b^	13.70±3.31^b^	1.4±0.39^c^	0.93±0.18^c^	34.5

a P<0.01, vs. normal group;

b P<0.001,

c P<0.01 and

d P<0.05 vs. placebo group.

MDD, score and lung index are presented as the mean ± standard deviation. MDD, mean time to death.

**Table II:** Effects of baicalin on the mRNA expression of TLR7 and MYD88 (mean ± standard error; n=8).

		2^−ΔΔCT^	
		
Group	Dose, mg/kg/day	TLR7	MYD88
Normal	-	1.01±0.15	1.03±0.13
Placebo	-	4.66±0.65^a^	3.27±0.53^a^
Ribavirin	100.00	2.39±0.41^b^	1.82±0.34^b^
Baicalin	93.75	3.95±0.45^c^	2.86±0.52^c^
	187.50	1.95±0.28^b^	1.85±0.13^b^
	375.00	1.82±0.27^b^	1.97±0.24^b^

a P<0.01 vs. normal group;

b P<0.01 and

c P<0.05 vs. placebo group.

TLR7, toll-like receptor 7; MYD88, myeloid differentiation primary response gene 88.

**Table III:** Effects of baicalin on the expression of TNF-α, IL-1β and IL-6 (mean ± standard error; n=12).

Groups	Dose, mg/kg/day	TNF-α, pg/ml	IL-1β, pg/ml	IL-6, pg/ml
Normal	-	256.23±44.45	653.69±94.15	5989.71±634.16
Placebo	-	765.85±95.47^a^	1055.21±121.47^a^	9048.64±1150.62^a^
Ribavirin	100.00	281.62±42.16^b^	740.5±103.29^b^	7581.41±852.24^b^
Baicalin	93.75	583.74±73.41^c^	957.94±111.38^c^	8476.21±958.56^c^
	187.50	310.23±47.58^b^	766.19±102.56^b^	6785.64±758.82^b^
	375.00	261.10±41.86^b^	729.17±84.19^b^	6550.96±657.47^b^

a P<0.01 vs. normal group;

b P<0.01 and

c P<0.05 vs. placebo group.

TNF, tumor necrosis factor; IL, interleukin.

## References

[1] Romanowska M, Stefanska I, Donevski S, Brydak LB (2013). Infections with A(H1N1)2009 influenza virus in Poland during the last pandemic: experience of the National Influenza Center. Adv Exp Med Biol.

[2] Wan QF, Wang H, Lin Y (2013). Effects of quercetin on CDK4 mRNA and protein expression in A549 cells infected by H1N1. Biomed Rep.

[3] Le QM, Kiso M, Someya K (2005). Avian flu: isolation of drug-resistant H5N1 virus. Nature (London).

[4] Saito R, Li D, Suzuki H (2007). High prevalence of amantadine-resistance influenza a (H3N2) in six prefectures, Japan, in the 2005–2006 season. J Med Virol.

[5] Sharma U, Bala M, Saini R (2012). Polysaccharide enriched immunomodulatory fractions from *Tinospora cordifolia*(Willd) miers ax hook. f. & Thoms. Indian J Exp Biol.

[6] Evers DL, Chao CF, Wang X (2005). Human cytomegalovirus-inhibitory flavonoids: studies on antiviral activity and mechanism of action. Antiviral Res.

[7] Wan QF, Gu LG, Yin SJ (2011). Protection effect of baicalin on lung injury of mice infected with influenza FM1. Chin J Tradit Chin Med Pharm.

[8] Wan QF, Gu LG, Yin SJ (2011). Effect of baicalin on cell apoptosis FAS/FAS-L system of pneumonia mice lung tissue infected with FM1. Chin Pharmacol Bull.

[9] Kitamura K, Honda M, Yoshizaki H (1998). Baicalin, an inhibitor of HIV-1 production in vitro. Antiviral Res.

[10] Romero MR, Efferth T, Serrano MA (2005). Effect of artemisinin/artesunate as inhibitors of hepatitis B virus production in an ‘in vitro’ replicative system. Antiviral Res.

[11] Chu ZY, Chu M, Teng Y (2007). Effect of baicalin on in vivo anti-virus. Zhongguo Zhong Yao Za Zhi.

[12] Xu G, Dou J, Zhang L (2010). Inhibitory effects of baicalein on the influenza virus in vivo is determined by baicalin in the serum. Biol Pharm Bull.

[13] Livak KJ, Schmittgen TD (2001). Analysis of relative gene expression data using real-time quantitative PCR and the 2(-Delta Delta C(T)) method. Methods.

[14] Cook DN, Beck MA, Coffman TM (1995). Requirement of MIP-1α for an inflammatory response to viral infection. Science.

[15] Kawai T, Akira S (2008). Toll-like receptor and RIG-I-like receptor signaling. Ann NY Acad Sci.

[16] MacLeod H, Wetzler LM (2007). T cell activation by TLRs: A role for TLRs in the adaptive immune response. Sci STKE.

[17] Walsh KB, Teijaro JR, Zuniga EI (2012). Toll-like receptor 7 is required for effective adaptive immune responses that prevent persistent virus infection. Cell Host Microbe.

[18] Rahman I, Gilmour PS, Jimenez LA (2002). Oxidative stress and TNF-α induce histone acetylation and NF-κB/AP-1 activation in alveolar epithelial cells: potential mechanism in gene transcription in lung inflammation. Mol Cell Biochem.

[19] Ludwig S, Ehrhardt C, Neumeier ER (2001). Influenza virus-induced AP-1-dependent gene expression requires activation of the JNK signaling pathway. J Biol Chem.

[20] Adachi M, Matsukura S, Tokunaga H (1997). Expression of cytokines on human bronchial epithelial cells induced by influenza virus A. Int Arch Allergy Immunol.

[21] Qian C, Cao X (2013). Regulation of toll-like receptor signaling pathways in innate immune responses. Ann NY Acad Sci.

[22] Aldridge JR, Moselev CE, Boltz DA (2009). TNF/iNOS-producing dendritic cells are the necessary evil of lethal influenza virus infection. Proc Natl Acad Sci USA.

[23] Severa M, Fitzgerald KA (2007). TLR-mediated activation of type I IFN during antiviral immune responses: fighting the battle to win the war. Curr Top Microbiol Immunol.

[24] Tenoever BR, Ng SL, Chua MA (2007). Multiple functions of the IKK-related kinase IKKɛ in interferon-mediated antiviral immunity. Science.

[25] Nimmerjahn F, Dudziak D, Dirmeier U (2004). Active NF-κB signaling is a prerequisite for influenza virus infection. J Gen Virol.

[26] Kumar N, Xin ZT, Liang Y (2008). NF-κB signaling differentially regulates influenza virus RNA synthesis. J Virol.

